# Construct and Criterion Validity of the Postmenopause Sexuality Questionnaire–PMSQ

**DOI:** 10.1055/s-0041-1733910

**Published:** 2021-08-30

**Authors:** Maria José Ferreira Lima, Marilia Duarte Valim, Sebastião Freitas de Medeiros

**Affiliations:** 1 Department of Gynecology and Obstetrics, Hospital Universitário Júlio Muller, Cuiabá, MT, Brazil; 2 School of Nursing, Universidade Federal de Mato Grosso, Cuiabá, MT, Brazil; 3 Department of Gynecology and Obstetrics, Faculdade de Ciências Médicas, Universidade Federal de Mato Grosso, Cuiabá, MT, Brazil

Dear Editor,

In 2020, (RBGO 42:26-32,2020) we have published the article “Construct and criterion validity of the postmenopause sexuality questionnaire—PMSQ” (DOI: https://doi.org/10.1055/s-0040-1701461 ). Complete copies, in English and Portuguese, of the instrument were attached to be published as supplemental material. Nevertheless, the instrument was not published. Since we have been asked to send a copy of the questionnaire to some readers of RBGO, we ask you, if possible, to publish both versions of the instrument in RBGO as supplemental material of the article.

## Editor-in-Chief Reply to the Authors

Dear authors,

In response to your request, we are publishing, in the current issue of RBGO, the two versions (English and Portuguese) of the Postmenopausal sexuality questionnaire – PMSQ.

Complementary material is available.

Thank you for your contribution to RBGO.

**Marcos Felipe Silva de Sá**

Editor-in-chief RBGO

