# Supplementary material for: Construct and Criterion Validity of the Postmenopause Sexuality Questionnaire–PMSQ
Source: Rev Bras Ginecol Obstet. 2021 Aug 30;43(7):578. doi: 10.1055/s-0041-1733910 (PMC10302069; doi:10.1055/s-0041-1733910)
Supplement: Supplementary file 1 — Supplementary Material [file 10-1055-s-0041-1733910-s211606.pdf]

English version:

POSMENOPAUSAL SEXUALITY QUESTIONNAIRE - PMSQ

Before applying the questionnaire, explain to the patient the importance of answering all the questions and request that the answers are sincere and true. Explain the purpose of the study, making it clear that the questions are about the intimacy of her sex life during the last 6 months. If the patient agrees to participate, read to her clearly the informed consent form and ask her to sign it.

A- DEMOGRAPHIC INFORMATION

Select the option that corresponds to your skin color
( ) White ( ) Black ( ) Mixed race ( ) Indigenous ( ) Yellow

What is your marital status?
( ) Single ( ) Married ( ) Widow ( ) Separated with partner ( ) Separated without partner

What is your degree of education?
( ) Illiterate ( ) Primary incomplete ( ) Primary complete
( ) Secondary incomplete ( ) Secondary complete ( ) Higher incomplete ( ) Higher complete

What is your profession?

Date of birth _______/______/_______
What is your age?

Where were you born?

How old were you when you started menstruating?

How old were you when you stopped menstruating?

B - SELF IMAGE

1. I am still a sensual, attractive woman.

Strongly disagree	Disagree	Slightly disagree	Slightly agree	Agree	Strongly agree
☐	☐	☐	☐	☐	☐

2. I feel good about my body image.

Never	Very seldom	Sometimes	Frequently	Very frequently	Always
☐	☐	☐	☐	☐	☐

C - QUALITY OF SEXUAL LIFE

3. I feel unsatisfied with my sexual activity.

Strongly disagree	Disagree	Slightly disagree	Slightly agree	Agree	Strongly agree
☐	☐	☐	☐	☐	☐

4. I want to improve my sensuality.

Strongly disagree	Disagree	Slightly disagree	Slightly agree	Agree	Strongly agree
☐	☐	☐	☐	☐	☐

5. I am unhappy with my sexual activity.

Strongly disagree	Disagree	Slightly disagree	Slightly agree	Agree	Strongly agree
☐	☐	☐	☐	☐	☐

D - SEXUAL INTIMACY

6. I hug and caress my partner's body during intercourse.

Never	Very seldom	Sometimes	Frequently	Very frequently	Always
☐	☐	☐	☐	☐	☐

7. I like to caress the penis of my partner

Never	Very seldom	Sometimes	Frequently	Very frequently	Always
☐	☐	☐	☐	☐	☐

8. I get emotionally involved with my partner during sexual intercourse.

Never	Very seldom	Sometimes	Frequently	Very frequently	Always
☐	☐	☐	☐	☐	☐

(*Continued*)

 © 2021. Federação Brasileira de Ginecologia e Obstetrícia. All rights reserved.

(*Continued*)

9. I like to be caressed by my partner.

Strongly disagree	Disagree	Slightly disagree	Slightly Agree	Agree	Strongly agree
☐	☐	☐	☐	☐	☐

10. I am concerned whit my sexual life.

Never	Very seldom	Sometimes	Frequently	Very frequently	Always
☐	☐	☐	☐	☐	☐

11. I am satisfied with my sentimental life.

Strongly disagree	Disagree	Slightly disagree	Slightly Agree	Agree	Strongly agree
☐	☐	☐	☐	☐	☐

E - DESIRE

12. I get aroused just by thinking about sex.

Never	Very seldom	Sometimes	Frequently	Very frequently	Always
☐	☐	☐	☐	☐	☐

13. I think, fantasize, dream of having sex/making love.

Never	Very seldom	Sometimes	Frequently	Very frequently	Always
☐	☐	☐	☐	☐	☐

14. I really want to get sexually aroused.

Strongly disagree	Disagree	Slightly disagree	Slightly Agree	Agree	Strongly agree
☐	☐	☐	☐	☐	☐

15. I have sexual intercourse less frequently than I would like to.

Strongly disagree	Disagree	Slightly disagree	Slightly Agree	Agree	Strongly agree
☐	☐	☐	☐	☐	☐

F - IMPORTANCE OF SEXUAL LIFE

16. I do want to have sex.

Strongly disagree	Disagree	Slightly disagree	Slightly Agree	Agree	Strongly agree
☐	☐	☐	☐	☐	☐

17. I am not interested in sex.

Strongly disagree	Disagree	Slightly disagree	Slightly Agree	Agree	Strongly agree
☐	☐	☐	☐	☐	☐

18. I feel sexually cold.

Strongly disagree	Disagree	Slightly disagree	Slightly Agree	Agree	Strongly agree
☐	☐	☐	☐	☐	☐

G - AROUSAL

19. I get wet during intercourse.

Never	Very seldom	Sometimes	Frequently	Very frequently	Always
☐	☐	☐	☐	☐	☐

20. I want to have sex.

Never	Very seldom	Sometimes	Frequently	Very frequently	Always
☐	☐	☐	☐	☐	☐

21. I like to have sex/make love.

Never	Very seldom	Sometimes	Frequently	Very frequently	Always
☐	☐	☐	☐	☐	☐

22. I feel like having sex when I am caressed.

Never	Very seldom	Sometimes	Frequently	Very frequently	Always
☐	☐	☐	☐	☐	☐

 © 2021. Federação Brasileira de Ginecologia e Obstetrícia. All rights reserved.

(*Continued*)

23. I feel pleasure during sexual intercourse.					
Never	Very seldom	Sometimes	Frequently	Very frequently	Always
☐	☐	☐	☐	☐	☐
**H - ORGASM**					
24. I only get an orgasm with great effort.					
Strongly disagree	Disagree	Slightly disagree	Slightly agree	Agree	Strongly agree
☐	☐	☐	☐	☐	☐
25. It is difficult for me to get an orgasm.					
Strongly disagree	Disagree	Slightly disagree	Slightly agree	Agree	Strongly agree
☐	☐	☐	☐	☐	☐
26. I get an orgasm easily.					
Strongly disagree	Disagree	Slightly disagree	Slightly agree	Agree	Strongly agree
☐	☐	☐	☐	☐	☐
27. It is impossible for me to have an orgasm.					
Strongly disagree	Disagree	Slightly disagree	Slightly agree	Agree	Strongly agree
☐	☐	☐	☐	☐	☐
**I - SATISFACTION**					
28. The feeling of sex is good.					
Strongly disagree	Disagree	Slightly disagree	Slightly agree	Agree	Strongly agree
☐	☐	☐	☐	☐	☐
29. Sex makes me feel fulfilled.					
Strongly disagree	Disagree	Slightly disagree	Slightly agree	Agree	Strongly agree
☐	☐	☐	☐	☐	☐
30. I feel satisfied with sex.					
Strongly disagree	Disagree	Slightly disagree	Slightly agree	Agree	Strongly agree
☐	☐	☐	☐	☐	☐
31. Considering the frequency of sexual relations with my current partner, I am:					
Very unhappy	Unhappy	Slightly unhappy	Slightly happy	Happy	Very happy
☐	☐	☐	☐	☐	☐
32. I get easily aroused when I am touched.					
Strongly disagree	Disagree	Slightly disagree	Slightly agree	Agree	Strongly agree
☐	☐	☐	☐	☐	☐
33. I feel uncomfortable during sexual intercourse.					
Never	Very seldom	Sometimes	Frequently	Very frequently	Always
☐	☐	☐	☐	☐	☐
**J - INFLUENCE OF MENOPAUSE**					
34. The fact that I no longer menstruate increased the frequency of my sexual intercourse					
Strongly disagree	Disagree	Slightly disagree	Slightly agree	Agree	Strongly agree
☐	☐	☐	☐	☐	☐
35. As a result of menopause, I feel less willing to have sex.					
Strongly disagree	Disagree	Slightly disagree	Slightly agree	Agree	Strongly agree
☐	☐	☐	☐	☐	☐
36. How was your desire to have sex after menopause?					
Poor	Very low	Low	Not changed	Improved	Very improved
☐	☐	☐	☐	☐	☐
**Thank you for your cooperation and sincerity.**					

 © 2021. Federação Brasileira de Ginecologia e Obstetrícia. All rights reserved.

Portuguese version:

QUESTIONÁRIO PARA O ESTUDO DA SEXUALIDADE FEMININA APÓS A MENOPAUSA

Antes de aplicar o questionário, explicar à paciente a importância de responder todas as questões e pedir que as respostas sejam sinceras e verdadeiras. Esclareça o objetivo do estudo, deixando claro que as perguntas são sobre a intimidade de sua vida sexual durante os últimos 6 meses. Caso a paciente concorde em participar, leia para ela com clareza o termo de consentimento livre e esclarecido e peça que o assine.

A – INFORMAÇÕES DEMOGRÁFICAS

Marque a opção que corresponde à cor da sua pele
( ) Branca ( ) Preta ( ) Parda ( ) Indígena ( ) Amarela

Qual é o seu estado civil?
( ) Solteira ( ) Casada ( ) Viúva ( ) Separada com parceiro ( ) Separada sem parceiro

Qual é o seu grau de instrução?
( ) Analfabeto ( ) Fundamental incompleto ( ) Fundamental completo
( ) Médio incompleto ( ) Médio completo ( ) Superior incompleto ( ) Superior completo

Qual é a sua profissão?

Data de nascimento? Qual é a sua idade?
____/____/______

Onde você nasceu?

Quantos anos você tinha quando começou a menstruar?

Quantos anos você tinha quando parou de menstruar?

B – AUTOIMAGEM

1. Ainda sou uma mulher sensual, charmosa.

Discordo muito	Discordo	Discordo pouco	Concordo pouco	Concordo	Concordo muito
☐	☐	☐	☐	☐	☐

2. Sinto-me bem com a minha imagem corporal

Nunca	Muito difícil	Às vezes	Com frequência	Com muita frequência	Sempre
☐	☐	☐	☐	☐	☐

C – QUALIDADE DE VIDA SEXUAL

3. Estou insatisfeita com a minha atividade sexual.

Discordo muito	Discordo	Discordo pouco	Concordo pouco	Concordo	Concordo muito
☐	☐	☐	☐	☐	☐

4. Quero melhorar minha sensualidade.

Discordo muito	Discordo	Discordo pouco	Concordo pouco	Concordo	Concordo muito
☐	☐	☐	☐	☐	☐

5. Estou infeliz com a minha atividade sexual.

Discordo muito	Discordo	Discordo pouco	Concordo pouco	Concordo	Concordo muito
☐	☐	☐	☐	☐	☐

D – INTIMIDADE SEXUAL

6. Eu abraço e acaricio o corpo do meu parceiro durante a relação sexual.

Nunca	Muito difícil	Às vezes	Com frequência	Com muita frequência	Sempre
☐	☐	☐	☐	☐	☐

7. Eu gosto de acariciar o pênis do meu parceiro.

Nunca	Muito difícil	Às vezes	Com frequência	Com muita frequência	Sempre
☐	☐	☐	☐	☐	☐

8. Eu me envolvo emocionalmente com o meu parceiro durante a relação sexual.

Nunca	Muito raramente	Às vezes	Com frequência	Com muita frequência	Sempre
☐	☐	☐	☐	☐	☐

 © 2021. Federação Brasileira de Ginecologia e Obstetrícia. All rights reserved.

(*Continued*)

9. Eu gosto de ser acariciada pelo meu parceiro.					
Discordo muito	Discordo	Discordo pouco	Concordo pouco	Concordo	Concordo muito
☐	☐	☐	☐	☐	☐
10. Eu me preocupo com a minha vida sexual:					
Nunca	Muito raramente	Às vezes	Com frequência	Com muita frequência	Sempre
☐	☐	☐	☐	☐	☐
11. Eu estou contente com a minha vida sentimental.					
Discordo muito	Discordo	Discordo pouco	Concordo pouco	Concordo	Concordo muito
☐	☐	☐	☐	☐	☐
**E – DESEJO**					
12. Eu fico excitada só de pensar em sexo					
Nunca	Muito raramente	Às vezes	Com frequência	Com muita frequência	Sempre
☐	☐	☐	☐	☐	☐
13. Eu penso, fantasio, sonho em fazer sexo/amor.					
Nunca	Muito raramente	Às vezes	Com frequência	Com muita frequência	Sempre
☐	☐	☐	☐	☐	☐
14. Eu realmente desejo ficar excitada sexualmente.					
Discordo muito	Discordo	Discordo pouco	Concordo pouco	Concordo	Concordo muito
☐	☐	☐	☐	☐	☐
15. Eu tenho relação sexual com menos frequência do que desejo.					
Discordo muito	Discordo	Discordo pouco	Concordo pouco	Concordo	Concordo muito
☐	☐	☐	☐	☐	☐
**F – IMPORTÂNCIA DA VIDA SEXUAL**					
16. Eu tenho vontade de fazer sexo.					
Discordo muito	Discordo	Discordo pouco	Concordo pouco	Concordo	Concordo muito
☐	☐	☐	☐	☐	☐
17. Eu não tenho interesse em sexo.					
Discordo muito	Discordo	Discordo pouco	Concordo pouco	Concordo	Concordo muito
☐	☐	☐	☐	☐	☐
18. Eu me sinto sexualmente fria.					
Discordo muito	Discordo	Discordo pouco	Concordo pouco	Concordo	Concordo muito
☐	☐	☐	☐	☐	☐
**G - EXCITAÇÃO**					
19. Eu fico molhada durante a relação sexual.					
Nunca	Muito raramente	Às vezes	Com frequência	Com muita frequência	Sempre
☐	☐	☐	☐	☐	☐
20. Eu sinto vontade de ter relação sexual.					
Nunca	Muito raramente	Às vezes	Com frequência	Com muita frequência	Sempre
☐	☐	☐	☐	☐	☐
21. Eu gosto de fazer sexo/amor.					
Nunca	Muito raramente	Às vezes	Com frequência	Com muita frequência	Sempre
☐	☐	☐	☐	☐	☐
22. Tenho vontade de ter relação sexual quando sou acariciada.					
Nunca	Muito raramente	Às vezes	Com frequência	Com muita frequência	Sempre
☐	☐	☐	☐	☐	☐

(*Continued*)

 © 2021. Federação Brasileira de Ginecologia e Obstetrícia. All rights reserved.

(*Continued*)

23. Eu sinto prazer durante a relação sexual.					
Nunca	Muito raramente	Às vezes	Com frequência	Com muita frequência	Sempre
☐	☐	☐	☐	☐	☐
**H - ORGASMO**					
24. Eu só tenho orgasmo depois de muito esforço.					
Discordo muito	Discordo	Discordo pouco	Concordo pouco	Concordo	Concordo muito
☐	☐	☐	☐	☐	☐
25. É difícil eu ter orgasmo.					
Discordo muito	Discordo	Discordo pouco	Concordo pouco	Concordo	Concordo muito
☐	☐	☐	☐	☐	☐
26. Eu tenho orgasmo com facilidade.					
Discordo muito	Discordo	Discordo pouco	Concordo pouco	Concordo	Concordo muito
☐	☐	☐	☐	☐	☐
27. É impossível eu ter orgasmo.					
Discordo muito	Discordo	Discordo pouco	Concordo pouco	Concordo	Concordo muito
**I - SATISFAÇÃO**					
28. A sensação de sexo é boa.					
Discordo muito	Discordo	Discordo pouco	Concordo pouco	Concordo	Concordo muito
☐	☐	☐	☐	☐	☐
29. Eu me sinto realizada com o sexo.					
Discordo muito	Discordo	Discordo pouco	Concordo pouco	Concordo	Concordo muito
☐	☐	☐	☐	☐	☐
30. Eu tenho satisfação com o sexo.					
Discordo muito	Discordo	Discordo pouco	Concordo pouco	Concordo	Concordo muito
☐	☐	☐	☐	☐	☐
31. Tendo em conta a frequência das relações com o meu parceiro, eu estou:					
Muito descontente	Descontente	Pouco descontente	Pouco contente	Contente	Muitocontente
☐	☐	☐	☐	☐	☐
32. Quando recebo carinho, me excito facilmente.					
Discordo muito	Discordo	Discordo pouco	Concordo pouco	Concordo	Concordo muito
☐	☐	☐	☐	☐	☐
33. Eu sinto desconforto durante a relação sexual.					
Nunca	Muito raramente	Às vezes	Com frequência	Com muita frequência	Sempre
☐	☐	☐	☐	☐	☐
**J – INFLUÊNCIA DA MENOPAUSA**					
34. O fato de não menstruar mais aumentou a frequência das minhas relações sexuais.					
Discordo muito	Discordo	Discordo pouco	Concordo pouco	Concordo	Concordo muito
☐	☐	☐	☐	☐	☐
35. Com a menopausa, eu estou menos disposta para o sexo.					
Discordo muito	Discordo	Discordo pouco	Concordo pouco	Concordo	Concordo muito
☐	☐	☐	☐	☐	☐
36. Como ficou a sua vontade de ter relações sexuais depois da menopausa?					
Péssima	Muito ruim	Ruim	Não mudou	Melhorou	Melhorou muito
☐	☐	☐	☐	☐	☐
**Muito obrigado por sua colaboração e sinceridade.**					

 © 2021. Federação Brasileira de Ginecologia e Obstetrícia. All rights reserved.
